# The Endocannabinoid System in Neuropsychiatric Disorders: Mechanisms, Dysregulation and Therapeutic Potential

**DOI:** 10.3390/biomedicines14050968

**Published:** 2026-04-23

**Authors:** Timur Mušić, Tamara Lah Turnšek

**Affiliations:** 1 University Medical Centre Ljubljana, Zaloška cesta 7, 1000 Ljubljana, Slovenia; 2 National Institute of Biology, Večna pot 121, 1000 Ljubljana, Slovenia; tamara.lah@nib.si

**Keywords:** endocannabinoid system, cannabinoid receptors, anandamide, 2-arachidonoylglycerol, neuropsychiatric disorders, stress-related response, mood disorders, eating disorders, ECS gene polymorphism, ECS epigenetic modulation

## Abstract

The endocannabinoid system (ECS) is a fundamental regulator of brain and body homeostasis, integrating neural, immune, and stress-related signaling pathways. Dysregulation of ECS components, including cannabinoid receptors (CB1 and CB2), endocannabinoids such as anandamide (AEA) and 2-arachidonoylglycerol (2-AG), and their metabolic enzymes (FAAH and MAGL), has been increasingly implicated in the pathophysiology of neuropsychiatric disorders, including mood, anxiety, psychotic, stress-related, and eating disorders. Altered endocannabinoid signaling contributes to maladaptive stress responses, emotional dysregulation, and impaired synaptic plasticity, highlighting the role of the ECS as a core integrative mechanism. Therapeutic strategies targeting ECS, particularly through FAAH inhibition and the use of plant-derived cannabinoids, such as cannabidiol (CBD), show promise in restoring endogenous homeostasis while minimizing the adverse cognitive and affective effects associated with direct CB1 activation. ECS function and treatment response are further influenced by genetic polymorphisms in *CNR1*, *CNR2*, *FAAH*, and *MGLL*, as well as epigenetic mechanisms, including DNA methylation, histone modifications, and microRNA regulation. Despite these advances, clinical translation remains limited by interindividual variability, the complexity of ECS interactions, and the relatively small size of existing clinical studies. Future research integrating longitudinal clinical trials with multi-omics approaches is essential to support the development of evidence-based, personalized interventions. Overall, understanding ECS mechanisms and dysregulation provides a valuable framework for the development of targeted therapies in neuropsychiatric disorders.

## 1. Introduction

The endocannabinoid system (ECS) has emerged as a critical neuromodulatory network involved in maintaining central nervous system homeostasis. It consists of cannabinoid receptors, endogenous cannabinoids, and metabolic enzymes involved in their synthesis and degradation. The ECS plays an important role in synaptic transmission, emotional processing, stress response, and neuroplasticity [1]. Growing evidence indicates that its dysregulation contributes to various neuropsychiatric conditions, underscoring its relevance to mental disorders [2].

Mental disorders represent a major and growing global health burden, significantly contributing to disability, reduced quality of life, and socioeconomic burden. These conditions encompass a broad spectrum of disturbances in cognition, emotion, perception, and behavior, arising from complex interactions among genetic susceptibility, environmental factors, and neurobiological mechanisms [3]. Despite substantial advances in psychiatric research, the molecular underpinnings of many mental disorders remain incompletely understood. Neuropsychiatric conditions, including mood and psychotic disorders, are particularly severe and heterogeneous. Major depressive disorder (MDD), bipolar disorder (BD), and schizophrenia are typically chronic, characterized by recurrent or persistent symptoms and marked functional impairment [4,5]. Contemporary pharmacological treatments for mental disorders are largely based on the monoaminergic hypothesis, targeting serotonergic, noradrenergic, and dopaminergic neurotransmission. Although these approaches have demonstrated clinical efficacy, therapeutic outcomes remain suboptimal for a substantial proportion of patients [6,7,8].

The limitations of traditional monoaminergic models have prompted the exploration of alternative neurobiological frameworks in psychiatry. In this context, growing attention has been directed toward the ECS and its modulation by plant-derived cannabinoids, particularly Δ^9^-tetrahydrocannabinol (THC) and cannabidiol (CBD). These compounds exert their effects primarily through CB1 and CB2 receptors, therefore playing a prominent role in the modulation of neurotransmission. In addition to their action at CB1 and CB2 receptors, plant-derived cannabinoids also interact with Transient Receptor Potential Vanilloid (TRPV) channels and G protein-coupled receptors (GPRs), contributing further to the fine-tuning of cannabinoid signaling [9]. This broader receptor engagement has deepened the understanding of ECS complexity and facilitated the subsequent identification of endogenous eCBs in both humans and animals, as described below.

Epidemiological evidence has consistently linked cannabis use with an earlier onset of psychosis and an increased risk of schizophrenia. Early findings by Ksir et al. (2016) suggested that cannabis use may act as a prodromal marker, interacting with familial and genetic vulnerability to contribute to disease development [10]. More recent epidemiological and real-world evidence indicate that frequent use of cannabis, particularly during adolescence, represents a significant risk factor for the development of psychosis in susceptible individuals [9,10]. These observations further highlight the critical role of cannabinoid signaling in brain function and its potential involvement in neuropsychiatric disorders.

The review summarizes current insights into the mechanisms linking the ESC to selected neuropsychiatric disorders and discusses emerging ECS-based therapeutic strategies with potential relevance for future clinical applications in psychiatry.

## 2. Endocannabinoid System: Structure and Function

### 2.1. Discovering Key ECS Components

ECS is a lipid-based signaling network that contributes to the maintenance of physiological homeostasis, increasingly recognized as an important integrative regulator of neuromodulation, immune regulation, and metabolic balance [3]. Pioneering work of Raphael Mechoulam, beginning in the 1960s when Mechoulam and Gaoni first isolated and characterized THC, the primary psychoactive component of *Cannabis sativa* [11], led to one of the most significant advances in human physiology. Although CBD had previously been described by Roger Adams and colleagues in 1940, its structure, closely related to THC, was elucidated by Mechoulam and Shvo in 1963, classifying both compounds as cyclic terpenoids [12]. The identification of specific cannabinoid receptors in the late 1980s and early 1990s marked a major turning point in ECS research. The CB1 receptor was first identified in the central nervous system, followed by the discovery of the CB2 receptor, predominantly expressed in immune tissues [13,14]. These findings strongly suggested the existence of endogenous ligands for cannabinoid receptors, a hypothesis confirmed by the isolation of anandamide (AEA) in 1992 [15] and shortly thereafter, 2-arachidonoylglycerol (2-AG) [16].

Recent comprehensive reviews, including that by Șerban et al. (2025), highlight the ECS as an integrative system linking the nervous, immune and gastrointestinal systems, particularly within the microbiota–gut–brain axis [16]. Collectively, these discoveries established the ECS as a distinct, evolutionarily conserved lipid signaling system that is deeply integrated into multiple physiological processes. ECS consists of three principal components: cannabinoid receptors, endogenous ligands, and metabolic enzymes that regulate ligand availability and signaling duration [17,18]. The two main biologically specific receptors, CB1 and CB2, have distinct yet complementary physiological roles. CB1 receptors are located primarily on presynaptic terminals, whereas CB2 receptors are found mainly in postsynaptic sites. CB1 is among the most abundant G-protein-coupled receptors in the human brain, with high expression in regions involved in cognition, emotion, memory, and motor control, including the cortex, hippocampus, and basal ganglia [18]. It plays a key role in neuromodulation across glutaminergic, GABAergic, serotonergic, and dopaminergic systems [3,17], related to distinct psychiatric disorders. In contrast, CB2 receptors are expressed in microglia, astrocytes, and, to a lesser extent, in postsynaptic neurons, where they contribute to anxiolytic and antidepressant effects [18]. In peripheral tissues, CB2 is predominantly expressed in immune cells, where it regulates inflammatory responses, immune cell migration, and cytokine production, thereby contributing to immune homeostasis [3,14,18,19].

**Figure 1:**
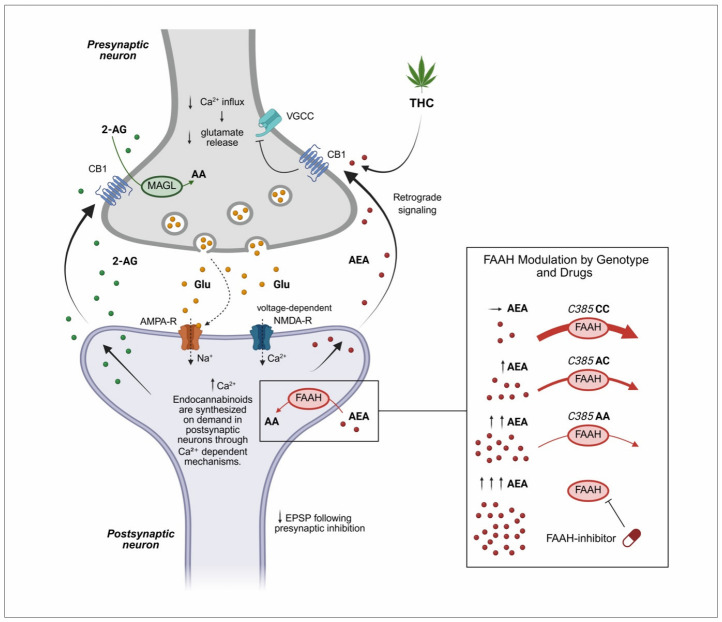
Endocannabinoid-mediated retrograde modulation of glutamatergic neurotransmission and FAAH polymorphism. Activation of the postsynaptic neuron induces Ca^2+^-dependent synthesis of endocannabinoids, including 2-AG and AEA. These molecules travel retrogradely across the synaptic cleft and activate CB1 on the presynaptic membrane, leading to inhibition of voltage-gated Ca^2+^ channels (VGCC) and suppression of glutamate release. Glutamate acts on ionotropic AMPA and NMDA receptors, mediating excitatory synaptic transmission and plasticity, which is reduced following presynaptic inhibition. THC, an exogenous CB1 receptor agonist, mimics endocannabinoid signaling. Endocannabinoid levels are regulated by FAAH and MAGL. Upward (↑) and downward (↓) arrows indicate relative increases or decreases in concentration, activity, or physiological effect, while dashed arrows denote modulatory or inhibitory influences, including feedback regulation and indirect effects. The inset illustrates the effect of the FAAH C385 (AA, AC, CC) polymorphism and pharmacological FAAH inhibition on AEA levels; the A allele is associated with reduced FAAH activity and increased AEA, with maximal levels observed under synthetic pharmacological FAAH inhibition. 2-AG—2-arachidonoylglycerol; AEA—anandamide; CB1—cannabinoid receptor type 1; VGCC—voltage-gated Ca^2+^ channels; Glu—glutamate; AMPA-R—α-amino-3-hydroxy-5-methyl-4-isoxazolepropionic acid receptor; NMDA-R—N-methyl-D-aspartate receptor; THC—Δ^9^-tetrahydrocannabinol; FAAH—fatty acid amide hydrolase; MAGL—monoacylglycerol lipase; EPSP—excitatory postsynaptic potential. Conceptually adapted from Tansey et al. (2025) [21]. Created in BioRender. Mušić T. (2026) https://BioRender.com/tf7mdik (accessed on 30 March 2026).

The primary endogenous cannabinoid ligands, AEA and 2-AG, are lipid-derived molecules that, although structurally distinct from phytocannabinoids such as THC and CBD, bind to the same CB1 and CB2 receptors, albeit with differing affinities [20]. Their localization and signaling are tightly regulated by their biosynthetic and degradative enzymes. Specifically, 2-AG is synthesized by diacylglycerol lipase (DAGL) and degraded by monoacylglycerol lipase (MAGL), whereas AEA is synthesized by the enzyme N-acyl phosphatidylethanolamine-specific phospholipase D (NAPE-PLD) and degraded by fatty acid amide hydrolase (FAAH). These processes regulate ligand levels in pre- and postsynaptic neurons, respectively (Figure 1). Importantly, the degradation of AEA in postsynaptic neurons is influenced by functional polymorphisms in FAAH, as illustrated in Figure 1, while 2-AG levels are affected by MAGL polymorphisms, which are discussed in Section 8.1.

### 2.2. Mechanism of ECS Action

A defining feature of the ECS signaling is that endocannabinoid (eCB) ligands are synthesized on demand from membrane phospholipid precursors rather than stored in vesicles, enabling rapid and spatially restricted responses to physiological stimuli [22,23]. eCBs (AEA, 2-AG) are synthesized through calcium-dependent mechanisms and released from postsynaptic neurons upon neuronal activation, traveling retrogradely across the synapse to activate presynaptic CB1 receptors (Figure 1) [23]. Activation of these receptors leads to inhibition of voltage-gated calcium channels (VGCC) and consequent reduction in glutamate release [24]. This prevents excessive excitatory drive in glutamatergic neurons and enhances transmission in inhibitory circuits, contributing to overall synaptic homeostasis [22,23,24]. The enzymatic activity of FAAH and MAGL regulates the eCB tone by influencing AEA and 2-AG levels [25], as also illustrated in Figure 1. In addition to FAAH, the FAAH family includes intracellular variants such as iFAAH, which localize to specific intracellular compartments, and FAAH2, a specific isozyme with broader substrate specificity and lower hydrolytic efficiency toward AEA [25]. Genetic variants in FAAH, FAAH2, and other related genes, including CNR1, CNR2, and MGLL, can influence enzyme activity and thereby eCB signaling [26]. Notably, FAAH2 variants, including missense and hemizygous mutations, have been linked to neuropsychiatric and metabolic phenotypes, highlighting a potential role in psychiatric disorders that is distinct from FAAH [27,28]. Together, these enzymes orchestrate AEA metabolism, regulating the duration, localization, and intensity of eCB signaling, with genetic polymorphisms contributing to interindividual variability in ECS function. Pharmacological inhibition of FAAH or MAGL may provide a strategy to restore eCB balance and mitigate dysregulation associated with genetic variability [21,29].

### 2.3. Major Physiological Functions of the ECS

The ECS functions as a dynamic homeostatic regulator that fine-tunes neurobiological responses to internal and external stimuli, supporting adaptive processes such as stress responsiveness, emotional regulation, learning, and memory formation [26,30]. By buffering excessive stress responses and modulating synaptic plasticity, the ECS contributes to behavioral flexibility and stress resilience. The ECS also exerts neuroprotective effects by limiting glutamate-induced excitotoxicity, primarily through the regulation of Ca^2+^ influx, thereby reducing oxidative stress and neuronal damage [24,30]. In addition, it modulates neuroinflammatory signaling by suppressing pro-inflammatory cytokine release and inhibiting nuclear factor kappa B (NF-κB) pathways, leading to reduced microglial activation [17]. Overall, the ECS is integral to the regulation of stress, fear, emotion, and social behavior. Its developmental dynamics [30,31,32,33] may contribute to vulnerability or resilience to psychiatric disorders, particularly during adolescence [26,31,32,33,34,35].

Beyond the central nervous system, the ECS coordinates immune and metabolic adaptations. It contributes to the resolution of inflammation, supports immune homeostasis, and integrates metabolic signals related to mitochondrial energy balance and nutrient availability [27]. Furthermore, eCB signaling acts as a bidirectional interface between peripheral physiology and brain function, particularly within the microbiota–gut–brain axis, integrating immune, metabolic, and neuroendocrine pathways [17,19]. Through these interactions, the ECS links neural, immune, and metabolic systems into a unified homeostatic network, supporting the development of cannabinoid-based and personalized therapeutic strategies.

## 3. Contemporary Models Linking the Endocannabinoid System to Psychiatric Disorders

Research on CB1 receptor biology has revealed dynamic developmental changes in receptor expression and distribution in both humans and animal models. CB1 expression becomes detectable around the 14th week of gestation, peaks during childhood, and stabilizes in adulthood. The highest receptor densities are observed in brain regions undergoing active maturation, including the prefrontal cortex, hippocampus, amygdala, basal ganglia, and cerebellum [30]. A major conceptual advance is the recognition of the developmental sensitivity of the ECS [31]. Early life and adolescence represent critical periods during which genetic variations, environmental stress, or exposure to exogenous cannabinoids can produce long-lasting effects on brain development and psychiatric risk [32]. Disruption in eCB signaling during these windows may interfere with synaptic pruning and circuit refinement, contributing to neurodevelopmental and adolescent-onset disorders [32], as also discussed in the next sections. Taking the above into account, there is a need for further research aimed at identifying more effective and targeted therapeutic strategies in the younger population [33,35].

Since the discovery of THC and the subsequent identification of cannabinoid receptors, the ECS has evolved from a pharmacological curiosity into a central framework for understanding brain function and psychiatric disorders [35]. Early observations of cannabis-induced alterations in mood, perception, cognition, and motivation provided the foundation for recognizing the ECS as an intrinsic neuromodulatory system [36,37]. Subsequent studies demonstrated that the ECS primarily stabilizes synaptic activity and neural network function rather than driving primary neurotransmission, highlighting its relevance in disorders characterized by network instability and dysregulated stress responses [3,17,37].

Contemporary models extend beyond receptor distribution to include molecular, genetic, and systems-level alterations of the ECS across psychiatric conditions. Dysregulation of eCB ligand levels, receptor expression, and metabolic enzyme activity has been reported in mood, anxiety, psychotic, and eating disorders [26,27,36,37]. Importantly, ECS dysfunction interacts with monoaminergic, glutamatergic, and neuropeptide-related systems [38]. These interactions reinforce the concept of the ECS as a cross-system integrator, with disorder-specific patterns of dysregulation that may vary across illness stages and clinical phenotypes [39,40]. Clinically, have simulated the development of both pharmacological and lifestyle-based strategies aimed at modulating eCB tone [41], as discussed in the following sections.

## 4. The Endocannabinoid System in Stress-Related Mental Disorders: Interaction with the HPA Axis

ECS plays a key modulatory role in the stress response through its interaction with the hypothalamic–pituitary–adrenal (HPA) axis. By regulating hypothalamic and pituitary activity, eCB signaling fine-tunes neuroendocrine stress responses, contributing to adaptive control of hormone release and maintenance of homeostasis under stress [42,43]. Stress or fear-inducing stimuli are initially processed in the amygdala, where threat perception is evaluated, and appropriate behavioral and physiological responses are initiated. Notably, eCB signaling during adolescence is particularly important for the regulation of HPA axis stress reactivity and the development of emotional behavior [44].

### 4.1. ECS Adaptive Control of Hormone Release

**Figure 2:**
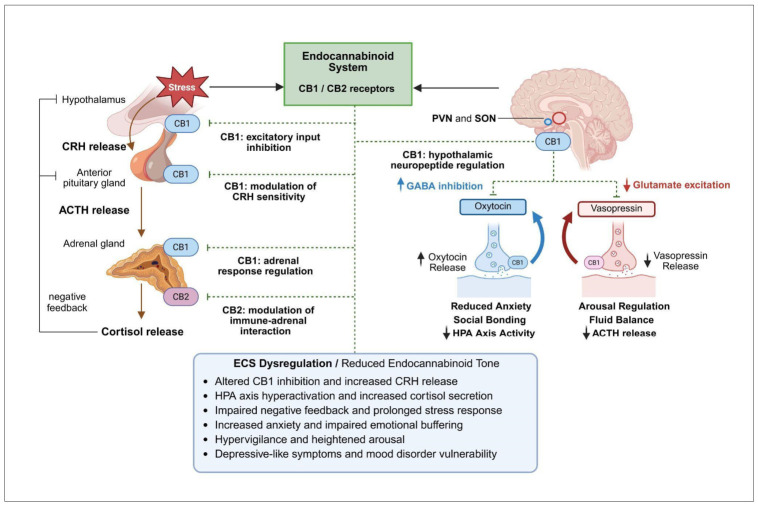
Schematic overview of stress processing and regulation of the HPA axis, highlighting modulation by the ECS and hypothalamic neuropeptides. Stress-related stimuli from the activated amygdala activate the PVN–CRH–ACTH–cortisol pathway, with cortisol exerting systemic effects and negative feedback. The ECS modulates HPA activity at multiple levels via CB1 signaling and, to a lesser extent, CB2 during prolonged stress. The second panel illustrates ECS interactions with oxytocin and vasopressin neurons in the PVN and SON, whereby CB1 activation facilitates oxytocin release and limits vasopressin-mediated stress amplification. Upward (↑) and downward (↓) arrows indicate relative increases or decreases in concentration, activity, or physiological effect, while dashed arrows denote modulatory or inhibitory influences, including feedback regulation and indirect effects. ACTH—adrenocorticotropic hormone; CB1—cannabinoid receptor type 1; CB2—cannabinoid receptor type 2; CRH—corticotropin-releasing hormone; ECS—endocannabinoid system; GABA—gamma-aminobutyric acid; HPA—hypothalamic–pituitary–adrenal; PVN—paraventricular nucleus; SON—supraoptic nucleus. Conceptually adapted from references [41,42,43,44,48,49,50]. Created in BioRender. Mušić T. (2026) https://BioRender.com/rstasrj (accessed on 18 February 2026).

Activation of the amygdala stimulates the paraventricular nucleus (PVN) of the hypothalamus to release corticotropin-releasing hormone (CRH), which in turn induces adrenocorticotropic hormone (ACTH) secretion from the anterior pituitary [45]. ACTH subsequently promotes glucocorticoid (cortisol) release from the adrenal glands. Cortisol mobilizes metabolic energy resources, suppresses inflammation, and enhances alertness, while negative feedback at the level of the hypothalamus and pituitary limits further HPA axis activation (Figure 2). In parallel, the ECS provides rapid, multi-level modulation of HPA axis activity. CB1 receptors signaling within the PVN suppresses excitatory input to CRH neurons, thereby constraining HPA initiation. At the pituitary level, CB1 activation modulates ACTH release by regulating responsiveness to CRH. Peripherally, CB1 signaling in the adrenal glands influences adrenal sensitivity to ACTH and glucocorticoid output. In addition, activation of CB2 receptors on immune cells may modulate immune–adrenal interactions during chronic stress. Collectively, eCB signaling regulates both the magnitude and duration of cortisol secretion [42,43,44,45].

The ECS is therefore a critical regulator of HPA axis activity and stress resilience [46,47]. Dysregulation of ECS–HPA interactions, due to reduced eCB tone or impaired receptor signaling, may result in prolonged glucocorticoid exposure and exaggerated stress responses, contributing to the pathophysiology of stress-related psychiatric disorders [48]. These include generalized anxiety disorder, post-traumatic stress disorder (PTSD), and MDD, all of which have been associated with altered HPA axis dynamics and eCB signaling, with early alterations often evident during adolescence [48]. Emerging evidence indicates that eCB dysregulation contributes to persistent HPA axis hyperactivity observed in clinical populations, supporting a mechanistic role of the ECS in the development of stress-related psychopathology [47,48]. Consequently, therapeutic strategies aimed at restoring ECS function and re-establishing balanced HPA axis regulation are gaining increasing attention. Preclinical and clinical studies suggest that modulation of ECS components can influence stress reactivity: exogenous cannabinoids such as THC and CBD exert distinct effects on HPA axis activity. THC demonstrates dose-dependent biphasic effects, whereas CBD appears to attenuate HPA hyperactivity, partly through indirect enhancement of endogenous AEA signaling [45,46,47,48]. Further mechanistic insights are required to support the development of targeted interventions addressing the underlying neurobiological dysfunction.

### 4.2. The Role of Neuropeptides in Stress Circuits

Recent evidence indicates that ECS closely interacts with hypothalamic-pituitary neuropeptides, particularly oxytocin and vasopressin, thereby extending its role beyond glucocorticoid feedback to broader modulation of neuropeptide-mediated stress circuits (Figure 2) [49]. Oxytocin and vasopressin are primarily synthesized in the paraventricular nucleus (PVN) and supraoptic nucleus (SON) of the hypothalamus and play important roles in social behavior, emotional regulation, and stress adaptation. Oxytocin is widely recognized for its anxiolytic, prosocial, and stress-buffering effects, particularly under conditions of acute stress. CB1 receptors are expressed on presynaptic terminals innervating oxytocin-producing neurons, where their activation suppresses inhibitory GABAergic input, thereby facilitating increased oxytocin release. Functionally, this enhances social bonding, reduces anxiety-like behavior, and promotes stress resilience by attenuating amygdala-driven fear responses and dampening HPA axis activity [50]. In contrast, vasopressin is associated with stress potentiation, increased social vigilance, and heightened HPA axis activation processes often dysregulated in stress-related psychiatric disorders, including anxiety disorders, PTSD, and MDD [51]. Vasopressin contributes to enhanced ACTH release and sustained stress responses. The ECS modulates this pathway through CB1 receptor-mediated inhibition of excitatory input to vasopressin neurons, thereby limiting excessive neuroendocrine activation [52,53]. Disruption of ECS–vasopressin interactions may therefore promote maladaptive stress reactivity, impaired emotional regulation, and reduced stress resilience, which are core features of stress-related psychopathology.

## 5. The Endocannabinoid System and Orexigenic and Anorexigenic Neuropeptide-Related Networks in Eating Disorders

**Figure 3:**
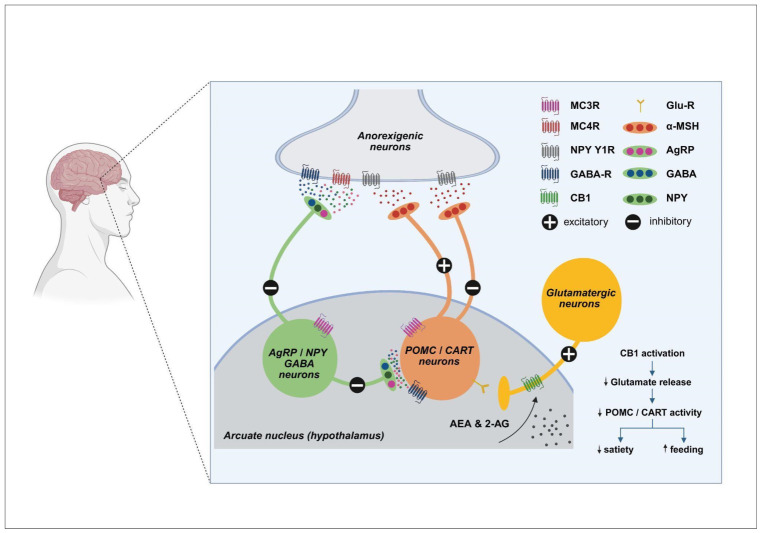
Schematic illustration of ECS regulation of hypothalamic feeding circuits in the arcuate nucleus. Anorexigenic POMC/CART neurons promote satiety via α-MSH release and activation of MC3R/MC4R in hypothalamic and brainstem regions, thereby reducing food intake and increasing energy expenditure. In contrast, orexigenic AgRP/NPY/GABA neurons inhibit POMC/CART activity and stimulate feeding. ECS signaling modulates this balance primarily through presynaptic CB1 receptors, where AEA and 2-AG suppress glutamatergic input onto POMC/CART neurons. This reduces α-MSH and CART output, attenuates satiety signaling, and functionally promotes feeding, particularly during energy deficit or acute stress. Dysregulation of ECS–POMC/CART interactions may contribute to maladaptive eating behaviors. Upward (↑) and downward (↓) arrows indicate relative increases or decreases in concentration, activity, or physiological effect, while dashed arrows denote modulatory or inhibitory influences, including feedback regulation and indirect effects. AEA—anandamide; 2-AG—2-arachidonoylglycerol; ECS—endocannabinoid system; POMC—pro-opiomelanocortin; CART—cocaine- and amphetamine-regulated transcript; α-MSH—α-melanocyte-stimulating hormone; MC3R—melanocortin receptor type 3; MC4R—melanocortin receptor type 4; AgRP—agouti-related peptide; NPY—neuropeptide Y; GABA—gamma-aminobutyric acid; CB1—cannabinoid receptor type 1. Conceptually adapted from [54,55,56]. Created in BioRender. Mušić T. (2026) https://BioRender.com/plpvvxk (accessed on 12 February 2026).

Accumulating evidence indicates that anorexia nervosa, bulimia nervosa, and obesity are associated with widespread alterations across all ECS components. Clinically, these disorders are characterized by high chronicity, frequent relapse, and limited efficacy of current pharmacological treatments, highlighting the need for novel therapeutic strategies [54]. The ECS has therefore emerged as a particularly attractive target due to its central role in the integration of appetite regulation, stress responsiveness, and reward processing. At the hypothalamic level, the ESC exerts critical inhibitory control over anorexigenic pathways mediated by pro-opiomelanocortin (POMC) neurons and cocaine- and amphetamine-regulated transcript (CART). POMC neurons, located primarily in the arcuate nucleus, release α-melanocyte-stimulating hormone (α-MSH), which activates melanocortin receptors (MC3/MC4) in downstream hypothalamic and brain stem nuclei to suppress food intake and increase energy expenditure. CART acts synergistically with POMC signaling to promote satiety and restrain stress-induced feeding (Figure 3) [54]. eCBs, particularly AEA and, to a lesser extent, 2-AG, modulate this system predominantly via CB1 receptor–mediated inhibition of excitatory synaptic input onto POMC/CART neurons. Activation of CB1 receptors reduces glutamatergic drive to these anorexigenic neurons, thereby decreasing α-MSH and CART release and functionally disinhibiting feeding behavior. This mechanism provides a rapid and reversible means of suppressing satiety signals during periods of energy deficit or acute stress. In pathological states, dysregulation of ECS–POMC/CART interactions can shift this balance toward maladaptive eating behaviors [55].

In anorexia nervosa, diminished eCB tone or impaired CB1 receptor signaling may result in insufficient inhibition of POMC/CART neurons, leading to persistent activation of anorexigenic pathways. This imbalance reinforces restrictive eating behaviors, exaggerated satiety signaling, and stress-related suppression of appetite. Reduced hypothalamic levels of eCBs, particularly AEA, may further exacerbate this imbalance by weakening CB1-mediated restraint of satiety circuits, thereby amplifying stress-induced appetite suppression and sustaining restrictive feeding patterns [56]. In contrast, obesity is often associated with chronic overactivation of the ECS, which can excessively suppress POMC/CART activity, attenuate satiety responses, and facilitate sustained hyperphagia [57,58]. Elevated circulating and central levels of eCBs, including 2-AG and AEA, appear to desensitize CB1-mediated anorexigenic signaling, thereby reinforcing overconsumption and contributing to the difficulty of achieving sustained weight loss [57]. Finally, in bulimia nervosa, unstable and dysregulated eCB modulation of POMC/CART circuits may drive the pathological cycling between restrictive eating and binge episodes, reflecting a failure to adequately integrate satiety signals, reward processing, and stress responsiveness [58].

From a clinical perspective, the ECS represents a promising therapeutic target in eating disorders, which exhibit distinct patterns of ECS dysregulation. In obesity, a condition of increasing prevalence in developed countries, chronic overactivation of ECS pathways, including enhanced CB1 signaling, may impair satiety mechanisms and reinforce maladaptive, reward-driven eating behaviors [58]. This is consistent with growing evidence suggesting that obesity may, at least in part, be conceptualized as a form of food addiction associated with ECS alterations. In this context, targeted modulation of FAAH or other enzymes regulating eCB tone may help restore homeostatic balance and attenuate compulsive food intake [58,59]. From both epigenetic and therapeutic perspectives, modulation of eCB tone, particularly via FAAH inhibition, provides a strategy to fine-tune hypothalamic POMC/CART activity and reward circuits without the adverse effects associated with direct CB1 agonism. Emerging evidence indicates that coordinated epigenetic dysregulation of *CNR1* and *FAAH* contributes to ECS dysfunction in anorexia nervosa [60], suggesting that disrupted eCB signaling is embedded within the disease’s molecular architecture rather than representing a secondary consequence. Context-dependent enhancement of endogenous AEA signaling may restore appropriate satiety responses, mitigate stress-driven feeding disturbances, and improve treatment responsiveness in both obesity and anorexia nervosa, conditions often resistant to conventional pharmacological approaches [55,56,59]. However, the psychoactive effects of direct CB1 agonists (e.g., phytocannabinoid THC) limit their clinical applicability, underscoring the therapeutic potential of targeting endogenous ECS modulation. Precision modulation of the ECS, attenuating overactivity in obesity or enhancing eCB tone in anorexia, offers a mechanism-based strategy for the treatment of eating disorders resistant to conventional therapies [60].

## 6. The Role of the Endocannabinoid System in Mood Disorders

Growing evidence supports a central role of the ECS in the regulation of mood, stress responsivity, and emotional homeostasis, positioning it as an important contributor to the pathophysiology of MDD and BD. Preclinical and clinical studies consistently demonstrate alterations in ECS components, including CB1 receptor signaling, circulating eCBs, and their metabolic enzymes, in individuals with mood disorders, particularly those exhibiting treatment resistance and pronounced stress sensitivity [38,39,61,62].

### 6.1. Major Depressive Disorder (MDD)

**Figure 4:**
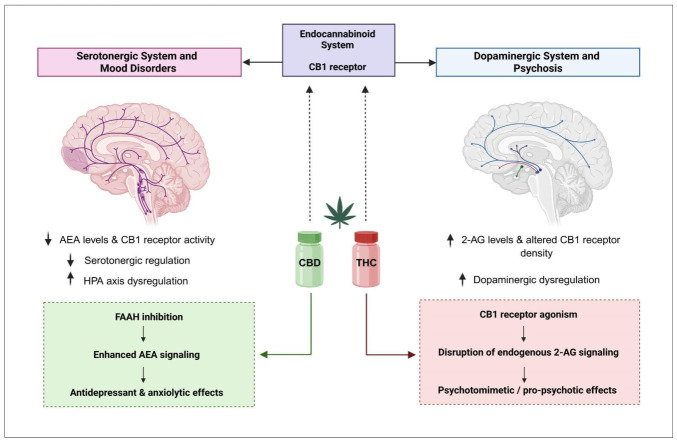
Endocannabinoid modulation of mood- and psychosis-related monoaminergic circuits. The ECS regulates serotonergic (**left**) and dopaminergic (**right**) pathways via CB1 receptors, thereby influencing mood, stress responsivity, neuroplasticity, and psychosis vulnerability. AEA and 2-AG modulate neurotransmission in both systems. Reduced AEA signaling and impaired CB1 function are associated with increased stress reactivity, anhedonia, and treatment resistance in MDD, whereas region-specific ECS alterations interacting with dopamine dysregulation contribute to psychosis. THC (CB1 agonist) may induce psychotomimetic effects and mood destabilization, while CBD (negative allosteric modulator of CB1 and indirect ECS regulator) exhibits anxiolytic and potential antidepressant and antipsychotic effects. Clinical outcomes depend on cannabinoid composition, dose, and individual susceptibility. Upward (↑) and downward (↓) arrows indicate relative increases or decreases in concentration, activity, or physiological effect, while dashed arrows denote modulatory or inhibitory influences, including feedback regulation and indirect effects. ECS—endocannabinoid system; AEA—anandamide; 2-AG—2-arachidonoylglycerol; CB1—cannabinoid receptor type 1; HPA axis—hypothalamic–pituitary–adrenal axis; THC—Δ^9^-tetrahydrocannabinol; CBD—cannabidiol. Conceptually adapted from [35,36,37,38,62,63,64,65,66,67,68,69,70,71,72,73,74,75,76,77]. Created in BioRender. Mušić T. (2026), https://BioRender.com/bwvi7mp (accessed on 20 February 2026).

Reduced AEA signaling and impaired CB1 receptor functions have been associated with increased stress reactivity, anhedonia, and dysregulation of the HPA axis in MDD [62]. These alterations interact closely with neuropeptide systems involved in emotional regulation, forming a complex network that modulates affective states [38,39,63,64,65]. However, ECS dysfunction is not uniform across patients, reflecting the marked biological heterogeneity of MDD and reinforcing the need for biomarker-driven, personalized therapeutic strategies [63,66]. Clinical evidence increasingly suggests that alterations of the ECS may represent a key biological substrate of treatment resistance in depression. Rosa et al. (2025) proposed that insufficient endogenous eCB tone may constrain the neuroplastic adaptations required for an adequate response to conventional antidepressants, thereby contributing to symptom persistence and incomplete remission [38]. From this perspective, pharmacological modulation of the ECS has emerged as a promising strategy to enhance antidepressant efficacy and address treatment resistance. Among available approaches, inhibition of FAAH represents a rational strategy to enhance AEA signaling without direct CB1 receptor overstimulation (Figure 4) [64,65]. Preclinical studies support antidepressant-like effects and synergistic interactions with monoaminergic agents. However, clinical translation has been limited by safety concerns related to off-target effects in early trials rather than by the mechanism itself [66]. CBD represents a clinically accessible alternative, exerting indirect ECS modulation through partial FAAH inhibition and allosteric modulation of CB1 receptors, alongside serotonergic effects [65,66]. Emerging clinical data suggest anxiolytic and antidepressant potential. However, heterogeneity in dosing and the limited number of controlled trials currently preclude definitive conclusions.

Other pharmacological strategies, including MAGL inhibition and CB1 allosteric modulation, remain largely experimental and raise concerns regarding excessive CB1 activation or insufficient clinical validation. Consequently, ECS-targeted pharmacotherapies have not yet been incorporated into standard treatment algorithms for depression. At present, aerobic physical exercise represents the most reliable and low-risk strategy for enhancing endogenous ECS activity [67]. Exercise may potentiate antidepressant efficacy by increasing circulating eCBs (AEA and 2-AG) that promote neuroplasticity and improve treatment outcomes, whereas remaining a non-pharmacological intervention with minimal adverse effects [68].

### 6.2. Bipolar Disorder (BD)

A particularly complex and clinically challenging context for ECS-based considerations is BD. Unlike MDD, BD is characterized by dynamic shifts between depressive and manic or hypomanic states, marked heterogeneity across subtypes, and a high burden of relapse and functional impairment [69]. These features complicate treatment strategies and raise specific concerns regarding interventions that may influence affective stability. Within this framework, ECS alterations appear to be state-dependent and closely linked with neuro-steroid signaling, inflammatory pathways, and stress responsivity, potentially contributing to mood instability and vulnerability to phase switching. Clinical and epidemiological data further indicate a high prevalence of substance use disorders in BD, particularly involving cannabis, which complicates the interpretation of ECS-related findings, especially in younger populations [70]. While exogenous cannabinoids, particularly THC, may transiently alleviate anxiety or depressive symptoms, their use is consistently associated with mood destabilization, increased cycling, and a higher risk of manic or mixed episodes [71,72]. This underscores a critical distinction between endogenous ECS modulation and exogenous cannabinoid exposure, emphasizing the need for caution when extrapolating ECS-based therapeutic strategies [73].

CBD has attracted greater interest than THC, due to its indirect ECS-modulating properties and favorable safety profile. Preliminary evidence suggests anxiolytic benefits and potential relevance in bipolar phenotypes with prominent stress sensitivity [72]. However, evidence for efficacy in core depressive or manic symptoms remains limited, and well-powered clinical trials are required. At present, standard mood stabilizers remain the cornerstone of treatment, whereas ECS-targeted interventions should be considered exploratory and adjunctive. Future precision approaches may aim to modulate ECS activity according to disease subtype and phase, with the goal of enhancing emotional resilience without triggering affective switching.

## 7. Pathophysiology of Psychosis and the Role of Endocannabinoids

Psychotic disorders are characterized by disturbances in perception, attribution, cognition, and affective regulation. These processes are strongly influenced by the ECS, which modulates dopaminergic signaling, cognitive function, and stress responsiveness [72,73,74]. Neurobiological studies consistently demonstrate altered ECS signaling in schizophrenia, including changes in CB1 receptor density and elevated levels of eCBs, particularly 2-AG, in cerebrospinal fluid and peripheral circulation (Figure 4). These alterations appear to interact with dopamine dysregulation in mesolimbic and prefrontal circuits, thereby influencing how the brain assigns importance to stimuli and contributing to psychotic symptom expression. Notably, ECS changes may precede the onset of disease, suggesting a role in vulnerability rather than merely reflecting chronic disease or medication effects [72,73].

The relationship between cannabis exposure and psychosis provides a clinically relevant framework for understanding ECS involvement. THC, the principal CB1 agonist, reliably induces dose-dependent, transient psychotomimetic symptoms in patients with psychotic disorders [72,75]. Longitudinal studies in first-episode psychosis indicate that persistent use of cannabis plant extracts is associated with poor functional outcomes and more severe symptom trajectories, reinforcing the concept of ECS-related vulnerability [74,75,76,77]. In contrast, CBD, acting as a negative allosteric modulator of CB1 receptors, appears to attenuate the harmful psychotomimetic effects of THC and may exert antipsychotic-like effects. Other beneficial CBD effects are likely mediated through indirect ECS modulation, including FAAH inhibition activity and consequent elevation of AEA levels, as well as interactions with serotonergic signaling and neuroprotective pathways. Importantly, the cognitive and psychosis-related effects of cannabis are highly context-dependent and influenced by the relative proportions of THC and CBD, individual susceptibility, and patterns of prior exposure [77]. A more detailed list of currently approved medical uses of phytocannabinoids is given in Section 8.3.

Evidence from real-world studies and pilot randomized controlled trials suggests opposing effects of THC and CBD on social cognition and psychosis-related outcomes, highlighting that cannabinoid composition, dose, and individual vulnerability are key determinants of clinical impact, rather than cannabis use *per se* [73,75,77]. This is consistent with experimental data demonstrating opposing CB1 receptor interactions and signaling effects of THC and CBD [16,17]. Clinically, these findings argue against simplistic therapeutic strategies based on CB1 activation and instead support a precision-based approach that distinguishes between modulation of endogenous ECS signaling and exposure to phytocannabinoids. Patients with schizophrenia exhibit altered expression of key ECS components, including AEA, 2-AG, and cannabinoid receptors [76]. These alterations are influenced by both prior cannabis exposure and antipsychotic treatment, underscoring the dynamic and context-dependent role of the ECS in shaping clinical presentation and therapeutic response [72,73,74,75,76,77]. Although CBD shows promise as an adjunctive treatment due to its indirect ECS modulation and favorable safety profile, evidence from randomized controlled trials remains limited, and its effects on core psychotic symptoms and cognition are not yet fully established [77], as further discussed in Section 8.

In summary, the ECS plays a dual role in psychosis, functioning both as a vulnerability factor and a modulator of symptom expression. Given the marked heterogeneity of psychotic disorders, careful patient stratification based on genetic profile, disease stage, and prior or current cannabis exposure is essential to minimize the risk of clinical destabilization and adverse outcomes.

## 8. Endocannabinoid System as a Therapeutic Target: Clinical Application, Genetic and Epigenetic Modulation

The ECS functions as a relevant integrator of neural, immune, and peripheral signaling, positioning it as a promising therapeutic target in complex disorders that are characterized by neuroinflammation and stress dysregulation. Many neuropsychiatric conditions share overlapping neuroimmune and inflammatory mechanisms that are tightly regulated by eCB interactions with specific cannabinoid receptors (CB1 and CB2) [78]. Dysregulation of ECS signaling, whether central or peripheral, may disrupt neuronal-immune communication, impair adaptive, and compromise stress regulation mechanisms increasingly implicated in the pathophysiology of anxiety, depression, and related disorders [78].

In specific psychiatric disorders, targeted enhancement of eCB signaling shows clinical relevance. In anxiety disorders, inhibition of FAAH increases AEA levels, thereby enhancing eCB tone and improving emotional regulation while avoiding direct CB1 stimulation due to psychomimetic risk [78,79]. CBD may further increase AEA levels, partially by impaired FAAH activity, but mainly by blocking fatty acid binding proteins transporting AEA to reach FAAH. These effects are associated with reduced hyperactivity of the amygdala and normalization of limbic circuitry during emotional processing tasks [79,80,81]. In stress-related disorders such as PTSD, altered CB1 receptor availability has been observed in the amygdala, hippocampus, and prefrontal cortex, reflecting disrupted eCB tone and contributing to impaired stress adaptation. Modulation of the ECS may facilitate fear extinction and reduce maladaptive fear memory processing, processes largely mediated by these regions [81,82]. Moreover, preclinical and translational evidence indicate that ECS modulation can facilitate fear extinction and reduce the retrieval and reconsolidation of fear memories, processes central to PTSD pathophysiology [82]. In mood disorders, including MDD and BD, dysregulated eCB signaling affects the prefrontal cortex, hippocampus, and nucleus accumbens, contributing to impaired stress adaptation, altered reward processing, and disturbances in emotional regulation [83]. Reduced AEA levels are associated with heightened HPA axis activation, while FAAH inhibition may restore stress-buffering capacity and improve mood, potentially alleviating anhedonia and affective instability [83,84,85]. In psychotic disorders, altered CB1 receptor availability and elevated AEA levels have been reported, whereas higher AEA concentrations are associated with lower symptom severity, suggesting a potential compensatory or protective role of eCB signaling. These findings support further exploration of ECS modulation as an adjunctive therapeutic strategy while avoiding direct CB1 stimulation due to psychomimetic risk [17,84]. Finally, in eating disorders, ECS-related alterations in the hypothalamus and mesolimbic reward pathways influence appetite, reward, and stress-driven responses. Enhancing eCB tone may help regulate stress-driven eating behaviors and reduce overeating, highlighting the therapeutic potential of ECS-targeted approaches in these conditions [86].

### 8.1. Genetic Polymorphisms of ECS Components: Implications for Treatment Response

#### 8.1.1. Genetic Polymorphisms in ECS Receptors

Genetic polymorphisms in ECS components related genes significantly influence receptor sensitivity, eCB metabolism, and downstream signaling dynamics (key variants are summarized in Table 1). 

Centered on the cannabinoid receptor genes CNR1 and CNR2, which are widely expressed in the brain, the ECS demonstrates pronounced regional and cellular specificity. Genetic variation in CNR1 may attenuate CB1 receptor responsiveness and impair stress-regulatory mechanisms, thereby increasing susceptibility to affective and stress-related disorders. In parallel, polymorphisms in CNR2, which influence immune and inflammatory signaling pathways, have been implicated in the pathophysiology of psychiatric conditions [87]. However, not all missense mutations are equally tolerated (as indexed by the probability of loss-of-function intolerance, pLI) [88], and functionally constrained genes within the ECS, such as *CNR1*, *DAGLA*, and *FAAH*, appear to preserve baseline signaling integrity, whereas *CNR2*, *MGLL*, and *FAAH2* exhibit greater tolerance to genetic variation, allowing for adaptive flexibility (Supplementary Table S1).

The *CNR1* gene has been extensively investigated in psychiatric genetics, including the characterization of its major isoforms. Among its variants, the rs1049353 polymorphism has not shown a consistent association with MDD in meta-analyses, suggesting that it is unlikely to represent a primary genetic risk factor [88]. However, accumulating evidence indicates that rs1049353 may modulate symptom severity through interactions with environmental stressors, such as childhood trauma, influencing PTSD-related threat-processing phenotypes, including hypervigilance and intrusive memories [89,90]. The *(AAT)n* repeat polymorphism in *CNR1* has shown modest and population-dependent associations with schizophrenia subtypes, although robust replication is lacking. In addition, the rs12720071 SNP has been linked to variability in cognitive performance in schizophrenia and psychosis, with evidence of interaction with environmental factors such as cannabis exposure affecting neurocognitive outcomes [91].

Overall, *CNR1* variants are better characterized as modulators of symptom expression, cognitive function, and gene–environment interactions rather than primary genetic risk factors for psychotic disorders. These findings suggest that *CNR1* polymorphisms may influence psychosis-related phenotypes through modulation of CB1 receptor signaling and neural circuit function in stress, reward, and cognitive pathways, thereby contributing to inter-individual differences in symptom severity and vulnerability.

The *CNR2* gene has been increasingly investigated as a contributor to psychiatric disorders due to its role in neuroimmune signaling and stress regulation. The rs2501432 (Arg63Gln) polymorphism has been consistently implicated in mood disorders, with meta-analytic evidence indicating a modest but significant association with increased risk of MDD [92]. Another missense variant, rs41311993 (524C>A; Leu133Ile), has been associated with BD in case–control studies, suggesting that structural alterations of the CB2 receptor may influence disease susceptibility, although replication remains limited [93]. Additional variants, including rs2229579, have been examined in schizophrenia, indicating potential involvement of CB2 in broader psychopathology. However, findings are inconsistent and context-dependent [94]. Emerging evidence indicates that *CNR2* variants may interact with environmental stressors and other ECS-related genes to shape affective phenotypes, although these findings remain preliminary. While rs2501432 and rs41311993 show the most consistent association with mood disorders, their effects are modest and most likely driven by gene-environment and gene-gene interactions [92,93,94], limiting their utility as independent risk markers. More broadly, *CNR1* and *CNR2* polymorphisms appear to exert context-dependent, modulatory effects on symptom expression and neural function rather than serving as primary diagnostic predictors. However, they may hold translational relevance as a potential biomarker of response to eCB-targeting therapies, pending validation in large, well-characterized cohorts.

#### 8.1.2. Genetic Polymorphisms in ECS Metabolic Enzymes

Polymorphic variants contribute to inter-individual variability in symptom severity, treatment response, and adverse-effect profiles associated with ECS-targeted interventions (Table 2). From a clinical genetics perspective, variants in enzymes regulating eCB metabolism are particularly relevant for psychiatric phenotypes. The *FAAH* rs324420 polymorphism shows the strongest and most consistent evidence, especially in anxiety, mood regulation, and stress-related processes, as reducing FAAH activity leads to elevated AEA levels and altered neural circuit signaling involved in fear extinction and emotional regulation [95]. Rare *FAAH2* mutations, although uncommon, have been associated with combined neurological and psychiatric symptoms, highlighting the importance of rare coding variants in atypical neuropsychiatric presentations. In contrast, evidence for *MGLL* variants in psychiatric disorders remains limited and inconsistent, with studies suggesting modest or context-dependent associations with PTSD and mood phenotypes [96]. Similarly, *DAGLA* and *DAGLB* exhibit high mutational constraint (Supplementary Table S3), indicating functional importance in neurodevelopment and synaptic regulation. However, current human genetic evidence linking these genes to psychiatric disorders is sparse, with rare DAGLA variants primarily associated with seizures and developmental abnormalities rather than mood disorders [97].

Overall, while *FAAH* variants, particularly rs324420, demonstrate the most robust and reproducible associations in humans, evidence for *MGLL* and *DAGL* genes remains emerging. These findings suggest that genetic variation in eCB metabolism may modulate vulnerability or resilience to anxiety, PTSD, and mood disorders, primarily through subtle, context-dependent effects rather than strong direct risk contributions.

### 8.2. Epigenetic Influences on ECS Function and Treatment Outcomes

Beyond genetic variability, epigenetic mechanisms provide an additional dynamic layer of ECS regulation. Chronic stress has been shown to induce hypermethylation of the *CNR1* promoter, resulting in reduced CB1 expression, whereas hypomethylation observed in certain neuropsychiatric conditions may lead to receptor overexpression. CB1 activation has also been associated with increased histone acetylation at neuroprotective loci, including brain-derived neurotrophic factor (BDNF). Moreover, microRNAs such as miR-146a and miR-155 regulate ECS-associated inflammatory pathways, thereby integrating immune and stress responses [98]. Environmental factors further influence these epigenetic processes. Omega-3 polyunsaturated fatty acids, dietary polyphenols, and gut microbiota-derived metabolites interact with ECS regulatory pathways, dynamically shaping eCB tone across the lifespan [99] (Table 3). Clinically, dysregulated CB1 activity may induce anxiety, cognitive impairment, or psychosis-like symptoms in susceptible individuals, whereas CB2 activation generally exerts anxiolytic effects with a more favorable side-effect profile [99,100]. Accordingly, precise dosing, careful titration, and close clinical monitoring are essential to optimize outcomes in cannabinoid-based therapies. Interindividual variability in ECS signaling, shaped by genetic, epigenetic, environmental, and developmental factors, further influences treatment efficacy and tolerability. Importantly, developmental stage must also be considered, as ECS activity changes during brain maturation, potentially altering the risk–benefit profile of ECS-targeted interventions [100].

Although routine screening for genetic and epigenetic markers could refine diagnosis and guide personalized treatment strategies, its clinical implementation remains limited. Advances in multi-omics and predictive modeling are expected to facilitate precision ECS-targeted therapeutics in psychiatry, offering safer and more effective alternatives to direct cannabinoid use, which, despite potential benefits, carries well-documented risks.

### 8.3. Pharmacokinetics of ECS Regulation

Preclinical and clinical studies, including animal models, emphasize that eCB tone critically depends on the balance between eCB synthesis and degradation, highlighting these enzymes as key therapeutic targets in psychiatric disorders [101]. As described in Section 2, eCBs are synthesized on demand from membrane lipid precursors by NAPE-PLD and DAGL, act locally at cannabinoid receptors, and are rapidly degraded within spatially restricted intracellular compartments enriched in FAAH or MAGL, indicating tightly regulated kinetics.

In vitro, FAAH exhibits a Km for AEAC of approximately 25 µM, and a relatively low Vmax (~0.29 nmol/min/mg protein), reflecting moderate substrate affinity and slower catalytic turnover. In contrast, MAGL shows a lower Km (~9.7 µM) and a markedly higher Vmax (~52 nmol/min/mg protein), supporting rapid hydrolysis of 2-AG. In vivo, these kinetic differences translate into distinct signaling dynamics: AEA produces more sustained and spatially restricted effects, whereas 2-AG signaling is more transient but reaches higher peak concentrations due to its greater abundance. Notably, MAGL-mediated hydrolysis in brain tissue is approximately 100-fold more active than FAAH-mediated degradation, explaining the efficient clearance of 2-AG despite its higher basal level [102].

These findings indicate that enzymatic activity is a principal determinant of eCB tone, effectively acting as a “metabolic switch” for ECS signaling. Accordingly, pharmacological inhibition of these enzymes represents a promising, though still developing, strategy for psychiatric treatment, with additional investigation in anxiety, depression, and pain-related conditions. FAAH inhibition (e.g., URB957) selectively increases AEA levels without significantly affecting 2-AG, whereas MAGL inhibition (e.g., JZL184) results in substantial elevations of 2-AG (up to eightfold in vivo). Similarly, ABX-1431 (also known as elcubragistat) elevates 2-AG levels and has demonstrated central nervous system target engagement along with acceptable safety profiles in early-phase clinical trials [103].

Complementary insights are provided by structurally modified eCB analogs with increased resistance to enzymatic degradation. AEA analogs, such as methanandamide and R-methanandamide, exhibit reduced susceptibility to FAAH, resulting in prolonged signaling, while AM404 indirectly enhances AEA levels by inhibiting both uptake and degradation. Similarly, 2-AG analogs, such as 2-arachidonoyl glycerol ether, replace the labile ester bond with a more stable ether linkage, conferring resistance to MAGL and extending signaling duration [102,103]. These findings demonstrate that relatively small structural modifications can substantially alter eCB pharmacokinetics, shifting signaling from transient to more sustained activity. However, further studies are required to fully validate this hypothesis in vivo, and clinical application of synthetic inhibitors remains in the developmental phase, requiring translation into larger clinical trials.

### 8.4. Therapeutic Applications and Delivery of Cannabinoid-Based Interventions

#### 8.4.1. Clinical Use of Phytocannabinoids

Clinically, therapies targeting the ECS have been established and approved by major regulatory agencies, including the U.S. Food and Drug Administration (FDA) and the European Medicines Agency (EMA). Phytocannabinoids, particularly THC and CBD, exert their effects primarily through CB1 receptors in the central nervous system, modulating processes such as pain perception, nausea, appetite, and motor control. In addition to indirect CB1-related effects, CBD also influences CB2-mediated pathways and increases AEA levels by inhibiting its degradation via FAAH, thereby contributing to anti-inflammatory and neuroprotective effects [104]. Epidiolex^®^ (purified CBD; European pharmacopeia 3151) is indicated for severe pediatric epilepsies, where it significantly reduces seizure frequency. Nabiximol (Sativex^®^, THC:CBD 1:1) is approved for the treatment of multiple sclerosis-related spasticity and has shown efficacy in chronic and neuropathic pain. Additionally, synthetic Δ^9^-THC analogs, such as dronabinol and nabilone, are used to stimulate appetite in patients with AIDS or cancer-related cachexia [105]. Whereas these compounds exhibit therapeutic potential, including the reduction in withdrawal symptoms or modulation of neuropsychiatric processes, their overall efficacy in psychiatric disorders remains limited and context-dependent, largely due to a lack of large-scale clinical trials and substantial interindividual variability in treatment response. Moreover, targeting the ECS *via* CB1 receptors may be associated with significant adverse effects, as outlined in the *Introduction*. Notably, the CB1 antagonist rimonabant, previously approved by the EMA for the treatment of obesity, demonstrated efficacy in promoting weight loss and improving metabolic parameters. However, it was subsequently withdrawn from the market due to severe psychiatric side effects, including depression and increased risk of suicide [106].

#### 8.4.2. Delivery Strategies for Endocannabinoid-Based Therapeutics in Psychiatric Disorders

The clinical application of eCB ligands, including AEA and 2-AG, is limited by their rapid enzymatic degradation, low aqueous solubility, and poor chemical stability [107]. To address these challenges, nanotechnology-based delivery systems have been developed, including lipid-based nanoparticles that encapsulate lipophilic eCB and enable controlled release. By protecting AEA and 2-AG from degradation by FAAH and MAGL, these systems improve pharmacokinetic stability and enhance therapeutic efficacy. Liposomal carriers facilitate cellular uptake *via* membrane fusion, while solid lipid nanoparticles stabilize the cargo within a solid lipid matrix, thereby prolonging systemic circulation compared to free eCBs [107,108].

In addition, chemical modification strategies have been employed to improve ligand stability, such as the development of prodrugs (e.g., esterified AEA analogs) that exhibit increased resistance to enzymatic hydrolysis. These approaches are often combined with carrier-mediated transporters, including nanoparticle surface functionalization with polyethylene glycol, albumin conjugation, or biomimetic coatings (e.g., cell membrane- or lipoprotein-based systems), which enhance solubility, prolong half-life, and facilitate transport across biological barriers [108]. Among emerging platforms, extracellular vesicles, particularly exosomes, represent highly promising delivery vehicles due to their intrinsic biocompatibility, targeting capacity, and ability to cross the blood–brain barrier [108,109]. Current research increasingly emphasizes precision-targeted approaches, including biologically derived carriers and combinatorial strategies that integrate eCB delivery with modulation of metabolic enzymes (FAAH, MAGL) and receptor-specific targeting, which may potentially optimize therapeutic outcomes in psychiatric disorders [109]. Additionally, accumulating evidence highlights the therapeutic relevance of CB2 receptor modulation in neuropsychiatric conditions, suggesting its potential as a complementary target to CB1-focused approaches [110]. Overall, converging preclinical and clinical evidence supports a significant role of eCBs and their metabolic enzymes in the pathophysiology of psychiatric disorders. Yet, at present, no large-scale clinical trials have definitively demonstrated the efficacy of these approaches, as most clinical studies have focused on neurological rather than psychiatric indications in larger populations.

## 9. Limitations and Future Directions in Endocannabinoid Research

This narrative review provides a focused overview of the therapeutic potential of the ECS in psychiatric disorders, particularly mood, psychotic, stress-related, and eating disorders. However, its scope is inherently limited by its narrative design, which precludes systematic quantitative synthesis or meta-analytic evaluation of treatment effects. The focus is limited to selected psychiatric conditions and may also overlook other relevant ECS-mediated disorders. In addition, translation of preclinical findings and pilot clinical trials to larger population clinical studies remains incomplete. Current clinical evidence is limited by small sample sizes, methodological heterogeneity, and short follow-up periods, which reduces the reliability and broader applicability of findings on ECS modulation, including FAAH and MAGL inhibition, as well as CB1 and CB2 receptor activity influenced by phytocannabinoids, such as CBD [111].

A major limitation is the lack of large-scale, well-powered clinical trials capable of establishing robust efficacy, safety, and dose–response relationships. Interindividual variability driven by genetic and epigenetic factors further complicates interpretation, underscoring the need for prospective, multicenter studies to validate biomarkers and facilitate their translation into clinical practice [111,112], while minimizing adverse effects associated with phytocannabinoid exposure [113]. Standardization of phenotyping, biomarker assessment, and clinical endpoints is also required to enable reproducible and comparable results across studies [114]. Overall, despite promising preclinical and early clinical findings, a substantial translational gap remains. Future progress will depend on large-scale, harmonized multicentric studies and the integration of precision medicine approaches, including pharmacogenetics and multi-omics profiling, to optimize patient selection and minimize adverse effects associated with ECS-targeted therapies.

## 10. Conclusions

ECS represents a central homeostatic regulator integrating stress responsivity, emotional processing, and synaptic plasticity, and thus plays an important role in the pathophysiology of psychiatric disorders. Dysregulation of ECS signaling is mechanistically linked to alterations in the HPA axis, dopaminergic imbalance, and impaired neuroplasticity across stress-related, mood, eating, and psychotic disorders. These convergent mechanisms highlight the integrative role of coordinating neurotransmitter systems underlying psychiatric phenotypes. However, clinical translation remains limited by biological heterogeneity, state-dependent effects, and the narrow therapeutic window associated with CB1 receptor modulation. Future progress will depend on integrating genetic and epigenetic profiling of key ECS-related genes, including *CNR1*, *CNR2*, *FAAH*, and *MGLL*, alongside DNA methylation patterns and microRNA signatures, into stratified treatment models to account for interindividual variability in eCB tone and treatment response. Epigenetic modifications related to several environmental factors may further modulate ECS function and contribute to disease vulnerability. Indirect modulation strategies, such as FAAH inhibition or carefully titrated cannabinoid-based approaches, may restore ECS balance while preserving physiological regulation. Ultimately, ECS-based therapies require a precision psychiatry framework that integrates molecular, developmental, and environmental factors to identify appropriate patient subgroups and optimize treatment timing and dosing, thereby improving clinical outcomes.

## Figures and Tables

**Table 1:** Key polymorphisms in ECS-related receptor genes. Summary of genetic variants with the highest constraint within key ECS components genes, *CNR1*, *CNR2*. The listed variants comprise common single-nucleotide polymorphisms (SNPs), functional missense substitutions, regulatory variants, and repeat polymorphisms that have been investigated in association studies. Adapted and summarized from references [87,88,89,90,91,92,93,94].

Gene and Notable SNP Variant	Location/Change	Psychiatric Associations	Findings/Effects
*CNR1*–rs1049353	Exon 4; synonymous coding	MDD, PTSD, anxiety	Modulates symptom severity through gene-environment interactions (e.g., childhood trauma), without a consistent direct association with disease risk
*CNR1*–(AAT)n repeat	3′ regulatory region	Schizophrenia	Population-dependent association may influence transcriptional regulation
*CNR1*–rs12720071	Intronic; regulatory SNP	Schizophrenia	Associated with variability in cognitive performance; interacts with environmental factors (e.g., cannabis exposure) to influence neurocognitive outcomes
*CNR2*–rs2501432	Intronic variant	MDD, schizophrenia	May modulate immune and inflammatory signaling, contributing to disease susceptibility
*CNR2*–rs2229579 (Gln63Arg)	Exon 3; missense	MDD, anxiety, schizophrenia	Functional missense variant; the Arg allele is associated with altered receptor signaling and increased vulnerability
*CNR2*–rs41311993 (Leu133Ile)	Coding region; missense	Bipolar disorder	Missense variant with potential effects on receptor structure and function

Legend: Polymorphisms in *CNR1* and *CNR2* genes identified in patients with psychiatric disorders, with corresponding variant annotations and reported clinical associations. *CNR1* is predominantly expressed in neurons where its variants are implicated in the modulation of CB1 receptor signaling, cognitive processes, and stress-related pathways. In contrast, *CNR2* is primarily expressed in immune cells and glia, with its variants influencing immune signaling and neuroinflammation processes, thereby exerting indirect effects on mood regulation and psychosis-related phenotypes. Gene constraint metrics indicate a slightly higher intolerance to loss-of-function variation for *CNR1* (pLI = 0.01) compared to *CNR2* (pLI = 0.00), suggesting greater tolerance of genetic variation in *CNR2*. Overall, variants in both genes are more consistently associated with modulation of symptom severity and gene-environment interactions than with high direct genetic risk for psychiatric disorders. Abbreviations: MDD—major depressive disorder; PTSD—post-traumatic stress disorder; SNP—single nucleotide polymorphism.

**Table 2:** Genetic variation in endocannabinoid-metabolizing enzymes and their associations with psychiatric phenotypes. This table summarizes key polymorphisms in genes encoding endocannabinoid-metabolizing enzymes and their associations with psychiatric phenotypes. Variants in *FAAH* show the most consistent evidence, while data for *FAAH2*, *MGLL*, and *DAGLA/DAGLB* remain limited and emerging. Adapted and summarized from references [95,96,97].

Gene & Variant (SNP)	Location/Change	Psychiatric Associations	Findings/Effects of Metabolizing Enzymes
*FAAH* (rs324420, C385A)	Missense variant (Pro129Thr), chromosome 1	Reduced anxiety, lower stress reactivity, and increased impulsivity	Reduces FAAH stability and activity, leading to elevated AEA levels and enhanced CB1 signaling
*FAAH2* (e.g., rs147173444; p.Ala458Ser)	X-linked missense variant	Learning difficulties and broader neurological symptoms	Partially reduces FAAH2 function, impairing endocannabinoid degradation and lipid signaling
*FAAH2* (Trp392Ter)	Nonsense variant (premature stop codon)	Severe developmental and neuropsychiatric phenotypes	Likely causes loss of FAAH2 function, disrupting fatty acid amide metabolism and ECS balance
*MGLL* (MAGL variants)	Intronic and coding variants	Inconsistent associations; possible links to stress response and mood regulation	Encodes the primary 2-AG-degrading enzyme; variation may alter endocannabinoid tone, although evidence is limited
*DAGLA/DAGLB*	Regulatory and coding variants	Potential involvement in neurodevelopmental and psychosis-related phenotypes	Encode enzymes responsible for 2-AG synthesis; variation may affect synaptic signaling and plasticity; human data remains limited
*FAAH* (other variants)	Regulatory and coding variants	MDD and PTSD	Alter FAAH expression/activity, influencing AEA levels, stress resilience, and emotional regulation

Legend: Genetic variants in key enzymes regulating endocannabinoid metabolism and their reported associations with psychiatric phenotypes. *FAAH* and *FAAH2* are involved in the degradation of AEA, whereas *MGLL* hydrolyzes 2-AG, and *DAGLA/DAGLB* mediate its synthesis. Variants in these genes may influence endocannabinoid tone, synaptic signaling, and stress-related neurobiological pathways, thereby contributing to inter-individual variability in psychiatric symptoms and treatment response. Abbreviations: AEA—anandamide; 2-AG—2-arachidonoylglycerol; ECS—endocannabinoid system; SNP—single nucleotide polymorphism; PTSD—post-traumatic stress disorder.

**Table 3:** The key epigenetic alterations in ECS and related components. The table summarizes key epigenetic and environmental factors that modulate ECS function and influence stress response, neuroplasticity, and neuropsychiatric outcomes. Adapted and summarized from references [98,99,100].

Epigenetic Mechanism	Primary Target	Effect on ECS Signaling	Psychiatric Relevance
DNA hypermethylation	*CNR1* promoter	Decreased CB1 receptor expression	Anxiety disorders, MDD, stress vulnerability
Histone acetylation	*BDNF* and neuroprotective genes	Enhanced CB1-mediated neuroplasticity and neuroprotection	Mood regulation, stress resilience, and cognitive decline
Histone deacetylation	Pro-inflammatory genes	Indirect modulation of CB2-mediated neuroimmune signaling	Neuroinflammation associated with depression and stress-related disorders
miR-146a	Inflammatory signaling pathways	Attenuation of ECS-related neuroimmune activation	Chronic stress, mood disorders
miR-155	Immune response genes	Modulation of CB2-associated neuroinflammatory responses	Neuroinflammatory components of psychiatric disorders
Dietary omega-3 PUFAs	Endocannabinoid synthesis pathways	Increased availability of AEA and 2-AG	Mood stability, cognitive function, stress adaptation
Gut microbiota-derived metabolites	ECS regulatory genes	Context-dependent modulation of ECS tone	Stress resilience, anxiety, microbiota-related mood disturbances

Abbreviations: ECS—endocannabinoid system; CB1—cannabinoid receptor type 1; CB2—cannabinoid receptor type 2; CNR1—cannabinoid receptor 1 (gene); BDNF—brain-derived neurotrophic factor; miR—microRNA; PUFAs—polyunsaturated fatty acids; AEA—anandamide (N-arachidonoylethanolamide); 2-AG—2-arachidonoylglycerol.

## Data Availability

No new data were created or analyzed in this study. Data sharing is not applicable.

## Supplementary Materials

The following supporting information can be downloaded at: https://www.mdpi.com/article/10.3390/biomedicines14050968/s1, Table S1: Structured overview of (1) GO ID and term, gnomAD-relevant annotation context for key ECS genes (grouped by category); Table S2: The role and key GO biological processes of ECS components classification of ECS gene-specific notes (generalized); Table S3: Gene constraint (pLI) across endocannabinoid system components.

## Abbreviations

The following abbreviations are used in the manuscript text (alphabetical order):

2-AG	2-arachidonoylglycerol
ACTH	adrenocorticotropic hormone
AEA	anandamide
BD	bipolar disorder
BDNF	brain-derived neurotrophic factor
CART	cocaine- and amphetamine-regulated transcript
CB1	cannabinoid receptor type 1
CB2	cannabinoid receptor type 2
CBD	cannabidiol
CNR1	cannabinoid receptor 1 gene
CNR2	cannabinoid receptor 2 gene
CRH	corticotropin-releasing hormone
DAGL	diacylglycerol lipase
DNA	deoxyribonucleic acid
ECS	endocannabinoid system
eCB	endocannabinoid
EMA	European Medicines Agency
FAAH	fatty acid amide hydrolase
FDA	U.S. Food and Drug Administration
GABA	γ-aminobutyric acid
GPR	G-protein coupled receptor
HPA	hypothalamic–pituitary–adrenal axis
iFAAH	intracellular fatty acid amide hydrolase
MAGL	monoacylglycerol lipase
MC3	melanocortin receptor 3
MC4	melanocortin receptor 4
MDD	major depressive disorder
NAPE-PLD	N-acyl phosphatidylethanolamine-specific phospholipase D
NF-κB	nuclear factor kappa B
POMC	pro-opiomelanocortin
PTSD	post-traumatic stress disorder
PVN	paraventricular nucleus (of the hypothalamus)
RNA	ribonucleic acid
SON	supraoptic nucleus (of the hypothalamus)
THC	tetrahydrocannabinol
TRPV	transient receptor potential vanilloid
VGCC	voltage-gated calcium channels
α-MSH	α-melanocyte-stimulating hormone
