# Supplementary material for: The Endocannabinoid System in Neuropsychiatric Disorders: Mechanisms, Dysregulation and Therapeutic Potential
Source: Biomedicines. 2026 Apr 23;14(5):968. doi: 10.3390/biomedicines14050968 (PMC13203790; doi:10.3390/biomedicines14050968)
Supplement: Supplementary file 1 [file biomedicines-14-00968-s001.zip › biomedicines-4201583-supplementary.pdf]

Supplement

Review on endocannabinoid system (ECS) related genes GO, gnomAD terms and gene constrains

Table S1. Structured overview of (1) GO ID and term, gnomAD-relevant annotation context for key ECS genes (grouped by category)

Gene	GO_ID	Category	GO_term	Parent_GO Process
FAAH11	GO:0006508	BP	proteolysis	metabolic process
FAAH11	GO:0016787	MF	hydrolase activity	catalytic activity
FAAH	GO:0006629	BP	lipid metabolic process	metabolic process
FAAH2	GO:0016787	MF	hydrolase activity	catalytic activity
FAAH2	GO:0006629	BP	lipid metabolic process	metabolic process
MGLL1	GO:0004806	MF	monoacylglycerol lipase	lipase activity
MGLL	GO:0044242	BP	cellular lipid catabolic process	lipid metabolic process
DAGLA1	GO:0003846	MF	diacylglycerol lipase activity	lipase activity
DAGLA	GO:0006687	BP	glycerolipid metabolic process	lipid metabolic process
CNR1	GO:0004930	MF	GPCR activity	molecular transducer activity
CNR1	GO:0007186	BP	GPCR signaling pathway	signal transduction
CNR2	GO:0004930	MF	GPCR activity	molecular transducer activity
CNR2	GO:0006955	BP	immune response	biological regulation

Legend: GO ID and terms (hierarchical context); Categories: MF-Molecular Function: receptor activity; BP- Biological Process

Table S2. The role and key GO biological processes of ECS components classification of ECS gene-specific notes (generalized)

Gene	Role in ECS	Key GO Biological Processes
FAAH	Anandamide degradation	lipid metabolism, neurotransmitter regulation
FAAH2	Lipid amide degradation	lipid metabolism
MGLL	2-AG degradation	endocannabinoid catabolism
DAGLA	2-AG synthesis	synaptic signalling
CNR1	CNS receptor	neurotransmission, GPCR signalling
CNR2	Immune receptor	immune signalling, GPCR

Legend: The enzyme FAAH (FAAH1) functions as a fatty acid amide and serine hydrolase, participating in lipid metabolism, endocannabinoid catabolism, and the regulation of neurotransmitter levels; FAAH2 exhibits hydrolase and lipid amide hydrolase activity, contributing to lipid metabolism and degradation with predicted intracellular localization. MAGL (MGLL) acts as a monoacylglycerol lipase and hydrolase, regulating 2-arachidonoylglycerol catabolism, lipid metabolism, and endocannabinoid signalling, and is found in the cytosol and plasma membrane. DAGLA functions as a diacylglycerol lipase, driving 2-AG biosynthesis and mediating synaptic signalling, including retrograde endocannabinoid signalling, localized to the postsynaptic and plasma membranes. CNR1, a cannabinoid receptor and G protein–coupled receptor (GPCR), is involved in GPCR signalling pathways, synaptic transmission, and modulation of neurotransmitter release, predominantly at the plasma membrane and along neuronal projections. Similarly, CNR2 is a cannabinoid receptor with GPCR activity, participating in GPCR signalling, immune processes, and inflammatory responses, and is localized to the plasma membrane, dendrites, and neuronal cell bodies.

Constraint evaluation of ECS components

"Gene constraint" refers to how tolerant a gene is to variation in a population, meaning how intolerant a gene is to the damaging mutations, and "constraint pattern" refers to where and how that intolerance is distributed across the gene or gene set.

Table S3. Gene constraint (pLI) across endocannabinoid system components

Gene	Protein / Function	ECS Role	pLI	Constraint Level	Functional Interpretation
CNR1	CB1 receptor	Central receptor	≥ 0.9	Highly constrained	Essential for CNS signaling
DAGLA	Diacylglycerol lipase α	2-AG synthesis	≥ 0.9	Highly constrained	Critical for endocannabinoid production
FAAH 1	Fatty acid amide hydrolase (FAAH1)	AEA degradation	≥ 0.9	Highly constrained	Tight regulation of ECS tone
FAAH2	FAAH paralog	AEA degradation	≤ 0.1	Loosely constrained	Functionally redundant; tolerant to variation
MGLL	Monoacylglycerol lipase	2-AG degradation	0.1–0.5	Moderately constrained	Partial functional importance
CNR2	CB2 receptor	Immune receptor	≤ 0.1	Loosely constrained	Immune-modulatory; flexible

Legend: Function pLi close to 1 means highly constrained gene, likely essential; pLI close to 0 means zero tolerance to loss-of-function variants. "Constraint pattern" refers to the distribution of constraint along a gene or across a gene set.

CNR1, CB1 is major cannabinoid GPR in brain, (high pLi ≥ 09 Typically constrained in functional domains. Essential, some. variants are associated with neurological phenotypes.
CNR2, CB2 receptor (low pLi≤ 0.1) | More variation tolerated; less essential than CB1. *DAGLA* is key biosynthetic enzyme in 2-AG synthesis, often high constraint (high pLi ≥ 0.9)
FAAH / MGLL are hydrolytic Rnzymes that often show moderate constrain. Functional variants linked to lipid signalling and neurological traits (low pLi (≤ 0.1) | More tolerant; partial redundancy with FAAH |
FAAH2 human-specific variation patterns (absent in rodents). Functional variants linked to neurological phenotypes (low–moderate pLi ~0.1–0.5), more |tolerance as synthetic*DAGL*
